# Antimicrobial resistance genes of *Escherichia coli*, a bacterium of “One Health” importance in South Africa: Systematic review and meta-analysis

**DOI:** 10.3934/microbiol.2023005

**Published:** 2023-02-13

**Authors:** Tsepo Ramatla, Mpho Tawana, Kgaugelo E. Lekota, Oriel Thekisoe

**Affiliations:** Unit for Environmental Sciences and Management, North-West University, Potchefstroom, 2531, South Africa

**Keywords:** *Escherichia coli*, antibiotic resistance genes, One Health, South Africa

## Abstract

This is a systematic review and meta-analysis that evaluated the prevalence of *Escherichia coli* antibiotic-resistant genes (ARGs) in animals, humans, and the environment in South Africa. This study followed Preferred Reporting Items for Systematic Reviews and Meta-analyses (PRISMA) guidelines to search and use literature published between 1 January 2000 to 12 December 2021, on the prevalence of South African *E. coli* isolates' ARGs. Articles were downloaded from African Journals Online, PubMed, ScienceDirect, Scopus, and Google Scholar search engines. A random effects meta-analysis was used to estimate the antibiotic-resistant genes of *E. coli* in animals, humans, and the environment. Out of 10764 published articles, only 23 studies met the inclusion criteria. The obtained results indicated that the pooled prevalence estimates (PPE) of *E*. *coli* ARGs was 36.3%, 34.4%, 32.9%, and 28.8% for *bla_TEM-M-1_*, *amp*C, *tet*A, and *bla*_TEM_, respectively. Eight ARGs (*bla_CTX-M_*, *bla_CTX-M-1_*, *bla_TEM_*, *tet*A, *tet*B, *sul*1, *sul*II, and *aad*A) were detected in humans, animals and the environmental samples. Human *E. coli* isolate samples harboured 38% of the ARGs. Analyzed data from this study highlights the occurrence of ARGs in *E. coli* isolates from animals, humans, and environmental samples in South Africa. Therefore, there is a necessity to develop a comprehensive “One Health” strategy to assess antibiotics use in order to understand the causes and dynamics of antibiotic resistance development, as such information will enable the formulation of intervention strategies to stop the spread of ARGs in the future.

## 1. Introduction

*Escherichia coli* is an enteric bacterium that lives in the intestinal tracts of humans and warm-blooded animals as part of commensal variations [1]. Animals are important reservoirs for pathogenic *E. coli* O157:H7 strains, and majority of the illnesses in humans are linked to undercooked meat, contaminated meat, water or raw milk consumption containing these pathogenic strains [2]. There are different pathotypes of *E. coli* that are related to the pathogenicity potential based on the presence of colonization factors or production of toxins that cause a variety of diseases [3], of which the majority are difficult to treat [4]. Majority of these strains have been isolated in humans and animals [5], however, water sources are regarded as a major public health risk [6],[7]. In response to bacteria gaining resistance to commonly used antimicrobial drugs, the expression of antibiotic resistance genes (ARGs) in bacteria is becoming a significant issue for public health [8].

Antibiotic resistant bacteria and their resistance genes have emerged as a critical and growing problem in modern medicine [9]. Additionally, it is a growing global public health concern for both animals and humans [10]. Antimicrobials used in human medicine are also utilized in livestock for growth promotion, disease prevention and disease treatment, thereby increasing selection pressures on bacterial pathogens, as well as the risk of antimicrobial resistance (AMR) onset and dissemination [11]. Different antibiotics have been used to treat *E. coli* infections in animals and humans [12],[13]. Overuse of antibiotics is common in animal husbandry and aquaculture, as they are used as feed additives for disease prevention and growth stimulation [14]. Bacteria develop antibiotic resistance through genetic alterations or the acquisition of ARGs from the host or environment [15].

Surveillance systems are still not well established in many developing nations due to a lack of financial support for sampling, testing, equipment acquisition, and maintenance. In developed countries, antimicrobial resistance surveillance systems implement whole genome sequencing (WGS) as a genotypic tool to supplement phenotypic antimicrobial susceptibility testing [13].

The spread of bacterial antibiotic resistance and pathogenicity imposes a significant health and economic cost [16]. Different bacterial ARGs can become resistant to various antibiotics [17]. Tetracyclines (*tet*), sulphonamides (*sul*), *β*-lactams (*bla*), macrolides (*erm*), aminoglycosides (*aac*), fluoroquinolone (*fca*), colistin (*mcr*) and vancomycin (*van*) are among the classes of antibiotics to which bacterial pathogens can express resistance genes. Key enteric pathogens, such as *Klebsiella* spp., *Salmonella* spp. *E. coli*, *Vibrio cholerae* and *Shigella* spp. have demonstrated unfavorable trends in the development of multi-drug resistance (MDR) in the African region to almost all widely available antibiotics [18]–[20]. Despite the high volume of antibiotics used in South Africa, there is a scarcity of knowledge about the relevant ARGs with regard to humans, animals, and the environment. Therefore, this study was carried out to identify prevalence gaps, analyze, and summarize the pooled prevalence of ARGs from *E. coli* isolates by carrying out a systematic review and meta-analysis of published studies in South Africa.

## 2. Materials and methods

### 2.1. Search strategy

Databases, such as African Journals Online (https://www.ajol.info/index.php/ajol/), PubMed (https://www.ncbi.nlm.nih.gov/pubmed/), ScienceDirect (https://www.sciencedirect.com/), Scopus (https://www.scopus.com/) and Google Scholar (https://scholar.google.com/), were searched for English articles published between January 2000 and December 2021. Relevant articles from each database were imported directly into spreadsheet (Microsoft Excel® 2013). All publications, including antimicrobial resistance genes from *E. coli*, were searched using the following keywords: Antibiotic resistance AND Antibiotic AND drug resistance AND bacterial resistance AND multi-drug resistance AND antibiotic resistance genes AND *Escherichia coli* OR *E. coli* AND Human OR animal [beef OR poultry OR livestock OR cattle OR animal OR cows OR chickens OR pig] AND Environment AND South Africa, with the last search conducted on 18^th^ of December 2021. The articles were screened by their title and abstract, and relevant publications were included in this study.

### 2.2. Inclusion and exclusion criteria

Studies were included on the basis that they fulfilled the following inclusion criteria; names of authors, location, total number of isolates, availability of the full texts, studies conducted in South Africa, studies that investigated antibiotic resistance genes, and articles published in English only on antibiotic resistance genes in *E. coli*, conducted from January 2000 to December 2021. Studies were excluded if they were not undertaken in South Africa, were reviews, book chapters, dissertations/thesis and not published in English.

### 2.3. Data extraction and statistical analysis

To reduce the possibility of bias, one author (TR) extracted the data, and a second author examined and confirmed it. The data was extracted from all eligible studies following the inclusion and exclusion criteria described above.

To assess the relative risk, we included articles reporting the number of antibiotic resistance genes in this meta-analysis. Studies were grouped based on bacterial species (*E. coli*). All statistical analyses were carried out using Comprehensive Meta-analysis (CMA) Version 3.0 by Biostat (Englewood, NJ, USA). The 95% confidence interval (CI) and pooled prevalence estimates (PPE) were calculated. The data generated was visualized using forest plots. The Cochrane Q test was used to calculate Cochran's heterogeneity (Q) among the included studies, as well as the percentage inverse variation (I^2^). If I^2^ was ≤ 25%, 50% or ≥ 75%, then heterogeneity was classified as low, moderate or high, respectively. The publication bias was assessed using funnel plots with ocular examination, including the Egger's and Begg's bias indicator tests. A random-effects model was used to generate all pooled estimates. Heterogeneity with a *P* < 0.05 were considered statistically significant.

## 3. Results

### 3.1. Literature search and eligible studies

**Figure 1.**
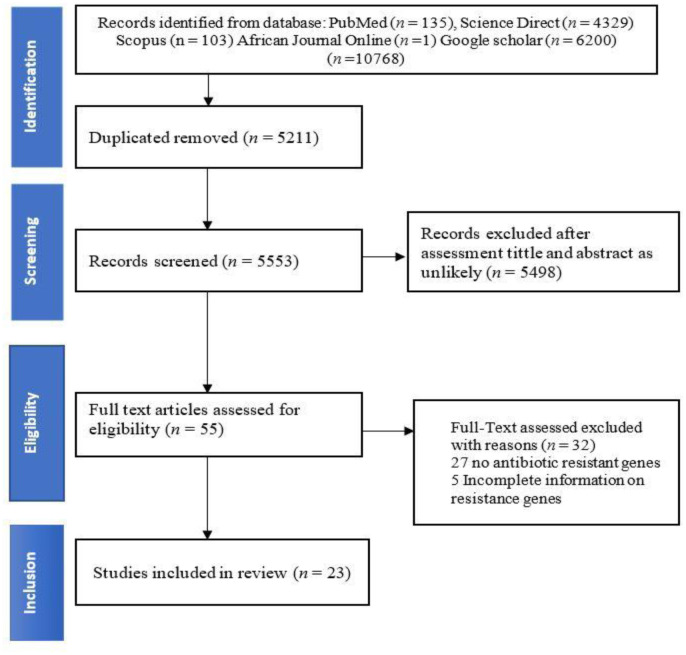
PRISMA flowchart showing selection of eligible articles for inclusion in this systematic review and meta-analysis of *Escherichia coli* antibiotic resistance genes in South Africa.

An electronic search of the databases African Journals Online, PubMed, ScienceDirect, Scopus, and Google Scholar yielded a total of 10764 articles (Figure 1). The search for articles related to studies on antibiotic resistance genes of *E. coli* in South Africa which were conducted throughout until December 2021. Duplication resulted in the removal of 5211 articles. Then, 5498 were excluded after the screening of titles, abstracts and languages. We evaluated 55 full-text papers for eligibility, and 32 of them did not meet our requirements. The exclusion was based on no reporting of the antibiotic resistance genes (n = 27) and incomplete information on resistance genes (n = 5). Only 23 peer-reviewed journal articles met the inclusion criteria. Table 1 summarizes studies that were included in this review with characteristics, such as province, method of detection, source of samples, number of isolates, and screened ARGs.

**Table 1.** Characteristics of eligible articles consisting of province, method of detection, source of samples, number of isolates and screened ARGs.

Reference	Province	Method used	Source of samples	One health segment	No. isolates	Antibiotic Resistance Genes
[21]	Eastern Cape	PCR	Wastewater treatment	Environment	223	*str*A, *aad*A, *cat* I, *cmlA*1, *bla*_TEM_, *tet*A, *tet*B, *tet*C, *tet*D, *tet*K, and *tet*M.
[22]	KwaZulu-Natal	m-PCR	Wastewater treatment plant	Environment	75	*bla_CTX_*_-M,_ *bla_TEM_*, *bla_KPC_*-2, *bla_OXA_*-1, *bla_NDM_*-1
[23]	North West	PCR	Humans, cattle, and pigs	Human and animal	76	*tet*B
[24]	Gauteng	PCR	Apples, carrots, tomatoes, spinach, and cabbage	Environment	56	*bla_TEM_*, *tetA*, *tetB*, *tetL*, *sulI*, *sulII*, *aadA1a*, *strAB*
[25]	North West	WGS	Faecal (beef and/or dairy)	Animal	80	*tet*A, *tet*B
[26]	North West	PCR	Stool samples from Human and water	Environment and human	212	*bla_CTX-M_*, *bla_DHA_*, *bla_SHV_*
[27]	KwaZulu-Natal	PCR	Wastewater treatment plants	Environment	80	*bla_CTX_-M*, *bla_TEM_*, *bla_SHV_*
[28]	Eastern Cape	PCR	Wastewater treatment plants	Environment	111	*mcr*-1, *erm*A
[29]	Eastern Cape	PCR	Faecal samples from dairy cattle	Animal	95	*blaampC*, *bla_CMY_*, *bla_CTX_-M*, *bla_TEM_*, *tet*A, *str*A
[30]	Eastern Cape	PCR	Irrigation water and agricultural soil	Environment	46	*tet*A, *tet*B, *tet*C, *cat*II, *cat*III, *sul*I
[31]	Eastern Cape	PCR	Carcasses	Animal	264	*aadA*, *strA*, *ampC*, *catI*, *tetB*, sul1.
[32]	KwaZulu-Natal	PCR	Urinary tract (Human)	Human	26	*bla_CTX_*-M, *gyr*A, *qnr*A, *qnr*B, *qnr*S, *qep*A*, aac (6′)-Ib-cr*
[33]	Gauteng	WGS	Human (blood, urine, and unknown sources)	Human	20	*bla_CTX_*-M, *bla_TEM_*-1B, *bla_OXA_*, *bla_CTX_-M-15*, *bla_OXA_*, *bla_CTX-M_-14*, *bla_CTX_-M-27* (E013), *bla_OXA_-10*, *blaCMY-2*
[34]	KwaZulu-Natal	PCR	Chickens (slaughter and final retail product)	Animal	266	*bla_CTX_-M*, *sul*1, *tet*A, *tet*B
[35]	Eastern Cape	PCR	Human (stool)	Human	265	*sul*II, *amp*C, *bla_TEM_*, *tet*A
[36]	North West	PCR	Cattle faeces	Animal	73	*aad*A, *str*A, *str*B, *erm*B, *tet*A
[37]	Eastern Cape	PCR	Stool samples from Human	Human	324	*ampC*, *bla_TEM_*, *sul*I, *sul*II, *aad*A, *tet*A.
[38]	Western Cape	PCR	Human	Human	12	*mcr*-1
[39]	Western Cape	PCR	Water from the river	Environment	171	*aadA*, *Bla*
[40]	KwaZulu-Natal	m-PCR	Wastewater treatment plant	Environment	146	*bla_TEM_*, *bla_CTX_*-*M*
[41]	Eastern Cape	PCR	Stool samples from Human	Human	106	*cat*A1, *tet*A
[42]	Western Cape	PCR	Humans	Human	22	*bla_CTX_-M*, *bla_CTX_*-*M*-15, *bla_CTXM_*-14, *bla_CTX_*-*M-*3.
[43]	Western Cape	PCR	Wildlife and livestock species	Animal	35	*bla_CMY_*, *sul*1, *sul*2, *aad*A1, *tet*A, *tet*B.

WGS = Whole Genome Sequencing, m-PCR = Multiplex PCR

Of the 23 included studies, 7 were environmental samples, 6 were samples from animal sources, 8 were from human and 1 included both human and environmental samples. All the studies included in this review were derived from five provinces in South Africa. Eastern Cape (n = 8) had majority of the studies, followed by KwaZulu-Natal (n = 5), North West (n = 4), Western Cape (n = 2) and Gauteng (n = 1) with the least number of studies (Table 1). The most common method for determining the antibiotics resistance genes of *E. coli* isolated from all articles included in this systematic review and meta-analysis was PCR (19/23:82.6%), followed by multiplex PCR (2/23:8.7%) and WGS (2/23:8.7%).

### 3.2. Pooled prevalence estimates (PPE) of antibiotic resistance genes

The *bla_TEM-M-1_* gene was detected from *E*. *coli* isolates with a PPE of 36.3% (95% CI: 18.7–58.5), followed by *amp*C gene 34.4% (95% CI: 16.6–58.1), *tet*A 32.9% (95% CI: 17.1–53.7), *bla*_TEM_ 28.8% (95% CI: 18.8–41.5), *bla*_TEM-M_ 23.3% (95% CI: 7.6–44.1), *bla*_SHV_ 22.6% (95% CI: 3.3–71.7), *str*A 21.7% (95% CI: 4.2–63.3), *aad* 19.4% (95% CI: 9.1–36.8), *sul*1 15.8% (95% CI: 5.6–37.4), *tet*B 14.7% (95% CI: 8.5–24.2), *cat*1 14.0% (95% CI: 0.1–94.8) and *sul*II 11.9% (95% CI: 4.1–30.3). The rest of the PPE of ARGs is shown in Table 2. However, genes such as *bla_OXA-1_*, *cat*2, *tet*D, *tet*K, *tet*G, *tet*M, *bla_CMY-2_*, *dfrA7*, *str*A, *bla*_*pse1*_, *bla*_*amp*_C, ant (3″)-la, *qnr*-B, *qnr*-S, *erm*B, *bla_CTX-M_*, *bla_CTX-M-15_*, *bla_CTX-M-3_* and *bla_SHV-2_* were not included for meta-analysis due to the low number of studies. The forest plot depicts the point estimate for individual studies, reporting the presence of *amp*C, *aad*A, *bla*_TEM_ and *tet*A (Figure S1).

**Table 2.** Pooled prevalence rate and 95% CI of antibiotic resistance genes of *E. coli* species based on meta-analysis.

Antimicrobial agents	Number of studies	Number of isolates	% Prevalence (95% CI)	I^2^ (95% CI)	Begg and Mazumdar rank *P*-value
*strA*	4	126	21.7	(4.2–63.3)	0.49691
*cat1*	3	109	14.0	(0.1–94.8)	0.60151
*blaCTX-M*	5	85	23.3	(7.6–44.1)	1.0000
*blaSHV*	4	201	22.6	(5.6–37.4)	1.0000
*tetB*	7	147	14.7	(8.5–24.2)	0.65230
*ampC*	3	104	34.4	(16.6–58.1)	0.60151
*sulII*	5	87	11.9	(4.1–30.3)	1.0000
*blaCTX-M-1*	6	167	36.3	(18.7–58.5)	0.85098
*blaTEM*	9	323	28.8	(18.8–41.5)	0.53161
*tetA*	10	401	32.9	(17.1–53.7)	0.17971
*sul1*	6	111	15.8	(5.6–37.4)	0.57303
*aadA*	5	172	19.4	(9.1–36.8)	0.14164

**Figure 2.**
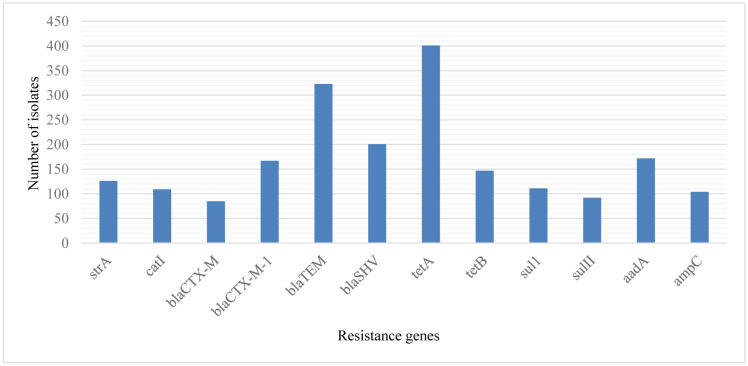
Antibiotic resistance genes detected in South African *E. coli* isolates from animals, humans, and the environment.

A total of 6 animal studies with 813 isolates were included in the meta-analysis, and they had a PPE of 25.4% (95% CI: 13.7–42.3) and 41.2% (95% CI: 10.1–81.4) for the *bla*_TEM_ and *tet*A genes, respectively. For humans, 8 studies with 738 isolates were included in this review. The *str*A gene had a PPE of 30.2% (95% CI: 4.2–81.1), followed by *tet*A 22.1% (95% CI: 9.1–44.7), *Sul*1 8.5% (95% CI: 6.5–11.1), *Sul*11 5.8% (95% CI: 2.9–11.4), and *tet*B 13.4% (95% CI: 10.9–16.2). While 7 studies from the environment were included in this review, only the *bla*_TEM_ gene was reported, with a PPE of 45.7% (95% CI: 22.5–70.9) from 685 isolates (Figure 2).

### 3.3. One health perspective

Of the 23 studies, 29 ARGs from humans, 26 from animals, and 19 from the environment were detected. Eight ARGs were detected in both humans, animals and in the environmental samples, whereas 9 were detected from humans and animals, 3 from animals and the environment and 2 from humans and the environment, as shown in Table 3.

**Table 3.** The antimicrobial-resistant genes (ARGs) detected between environmental, humans and animals.

Human & animal	Human & environment	Animal & environment	Animals, human & environment
*amp*C	*bla_SHV_*	*aad*A1a	*bla_CTX-M_*
*str*A	bla_OXA-1_	*aad*A1	*bla_CTX-M-1_*
*cat*I	*qnr*B	*erm*B	*bla_TEM_*
*cat*II			*tet*A
*cml*A1			*tet*B
*tet*C			*sul*1
*tet*D			*sul*II
*tet*M			*aad*A
*qnr*B			

### 3.4. Publication bias

The Begg and Mazumdar rank correlation test demonstrated no significant publishing bias for all parameters.

## 4. Discussion

Most of the studies included in this review were conducted on humans (34.8%). Out of the nine provinces, only five (55%) provinces, that is, North West, Eastern Cape, KwaZulu-Natal, Gauteng and Western Cape, were represented in this study. However, the Free State, Limpopo, Mpumalanga, and Northern Cape were not represented in the data sets, which may be due to a lack of research facilities in these provinces and/or a scarcity of researchers in the infectious microbiology field. The other reason might be that there are no Medical Research Council (MRC) institutes in those provinces.

AMR continues to increase internationally as a result of the widespread and unchecked use of antibiotics in veterinary and medical procedures [44]. Bacterial antibiotic resistance can spread to unaffected bacteria via DNA or other genetic components like integrons, bacteriophages and transposons [45]. Bacteria expressing ARGs are on the rise as a result of widespread agricultural practices, and the excessive and uncontrolled use of antibiotics to treat human illnesses [45]. Humans, animals, and the environmental components interact, and either directly or indirectly contribute to the spread of antimicrobial resistance [46],[44]. In this study, a high prevalence of ARGs in *E. coli* was found in both human and animal samples.

Twelve resistance genes, namely streptomycin (*str*A), chloramphenicol (*cat*I), *β*-lactams (*bla_TEM_*, *bla_CTX_*-*M*, *bla_CTX_-M*-1, *bla_SHV_*), sulphonamides (*sul1* and *sul*II), aminoglycosides (*aadA*), ampicillin (*ampC*) and tetracycline (*tetA* and *tetB*) were the most detected resistant genes, based on data obtained from studies analyzed in the current review. Infections brought on by pathogenic *E. coli* have been successfully treated with *β*-lactam antibiotics. However, a vast number of hydrolytic enzymes, namely the *β*-lactamases produced by bacteria, are currently seriously impairing the usefulness of *β*-lactams [47]. The *tet* (A, B, and C) gene is amongst detected genes in *E. coli* in this study from both animals, humans, and the environment. Tetracyclines are the most often used or overused antibiotics in livestock production in South Africa [48],[44]. Furthermore, Eagar et al. [49] indicated that tetracyclines were the most commonly used antibiotics in animals in South Africa between the years 2002 and 2004, hence, it is not surprising that most bacteria have a high level of tetracycline (*tet*) resistance [45],[50],[51]. Therefore, the excessive continued use of this antibiotic has led to the development of resistance. The chloramphenicol, *cat*I gene, was also detected in humans and animals. This is surprising because chloramphenicol has been removed from standard prescription lists due to the side effect of bone marrow aplasia. Gene cassettes of the *aad*A have been widely found in the environment and in animal production. The *aad*A group of genes encodes resistance to streptomycin and spectinomycin [14].

The quinolones, *qnr* gene, was also found in *E. coli* isolates of humans, animals and the environment in this study. DNA gyrase and topoisomerase IV are protected from quinolone chemicals by the genes (*qnr*) expressing proteins that are members of the pentapeptide repeat family, which mediates quinolone resistance in plasmids [52],[53]. According to this study, environmental organisms may have been the source of the circulating *qnr* genes [54]. Fluoroquinolone resistance is significant since it can spread rapidly among bacterial species that threaten human health. The cross-species and cross-genus transfers of resistance determinants are also possible [55].

In this review, three major molecular approaches were utilized to detect ARGs, such as PCR, multiplex PCR, and whole genome sequencing (WGS). Eighty-eight percent of the articles used traditional PCR techniques, most likely due to easy access to PCR cyclers and the reduced costs involved with PCR. Despite the fact that WGS offers a number of benefits, it was only utilized twice in all of the studies analyzed. More than 70 genes that may be related to drug resistance have been found in many recent large WGS investigations [56]. The WGS analysis has demonstrated the capacity to eliminate phenotypic and genotypic inconsistencies [56]–[58]. Due to its ability to quickly identify resistance pathways, WGS has become a crucial tool for profiling ARGs and has also played a role in measuring the rate at which resistance emerges [56]. WGS and other high-throughput diagnostic technologies have shown significant promise in medical diagnostics, and have proven to be essential in the control of antibiotic resistance [59].

Using the “One Health” approach, multiple disciplines work locally, nationally, and internationally to achieve optimal human, animal, and environmental health, realizing that the three are interconnected [60]. Since humans, animals, plants, food, and the environment are the main sources of antimicrobial resistance, the necessity of a “One Health” control strategy is highlighted in combating this problem [15]. The presence of similar zoonotic *E. coli* isolates, in animals, humans and the environment must be taken into consideration in South Africa. Food safety, zoonotic disease control, laboratory services, neglected tropical diseases, environmental health, and antimicrobial resistance are among the areas of work where a “One Health” approach is particularly relevant, according to the World Health Organization (WHO) (https://www.euro.who.int/en/home). The WHO recommends using a “One Health” approach to address health threats at all three interfaces [10],[62]. There is a dynamic interaction between human, animal, and environmental components that contribute to the rapid emergence and spread of antimicrobial resistance, either directly or indirectly [44]. This concept emphasizes the importance of balance and interconnectedness across the human-animal-environment sectors.

Even though we have organized data on the prevalence of antibiotic resistance genes in *E. coli*, the following limitations apply to our study: PPE of some resistant genes were not calculated because there are few reports on each. With respect to provinces, Limpopo, Free State, Mpumalanga, and Northern Cape are underrepresented.

## 5. Conclusions

This systematic review and meta-analysis gave an overview of scientific data on *E. coli* antibiotic resistance genes in human, animal, and environmental samples from South Africa. There are significant gaps in surveillance and a lack of published studies on the prevalence of *E. coli* resistance genes in some provinces like Limpopo, Free State, Mpumalanga, and Northern Cape. This study revealed the highest PPE of *E. coli* resistance genes to *amp*C, *tet*A, *bla*_TEM_, *bla*_TEM-M_, *bla*_SHV_, *str*A, *aad*, *sul*1, *tet*B and *cat*1, while eight genes (*bla_CTX-M_*, *bla_CTX-M-1_*, *bla_TEM_*, *tet*A, *tet*B, *sul*1, *sul*II and *aad*A) were detected in *E. coli* isolates from animals, humans, and the environment. This finding calls for the restricted use of this group of antibiotics. There is also a need for detailed studies that document the relationships between the phenotypic and genotypic occurrences of antibiotic resistance, as well as the presence of virulence genes. The fact that resistance genes have been detected in humans, animals, and environmental samples means there is a need for consolidated “One Health” approaches from the ecological, human, and animal health sectors in terms of epidemiological, therapeutics, and policy formulation research.
