# Supplementary material for: Antimicrobial resistance genes of Escherichia coli, a bacterium of “One Health” importance in South Africa: Systematic review and meta-analysis
Source: AIMS Microbiol. 2023 Feb 13;9(1):75–89. doi: 10.3934/microbiol.2023005 (PMC9988412; doi:10.3934/microbiol.2023005)
Supplement: Supplementary file 1 [file microbiol-09-01-005-s001.pdf]

AIMS Microbiology

AIMS Microbiology, 9(1): 75–89.
DOI: 10.3934/microbiol.2023005
Received: 20 December 2022
Revised: 07 February 2023
Accepted: 08 February 2023
Published: 13 February 2023

http://www.aimspress.com/journal/microbiology

Review

Antimicrobial resistance genes of *Escherichia coli*, a bacterium of "One Health" importance in South Africa: Systematic review and meta-analysis

Tsepo Ramatla*, Mpho Tawana, Kgaugelo E. Lekota and Oriel Thekisoe

Unit for Environmental Sciences and Management, North-West University, Potchefstroom, 2531, South Africa

* **Correspondence:** Email: ra21205450@gmail.com; Tel: +27182992521.

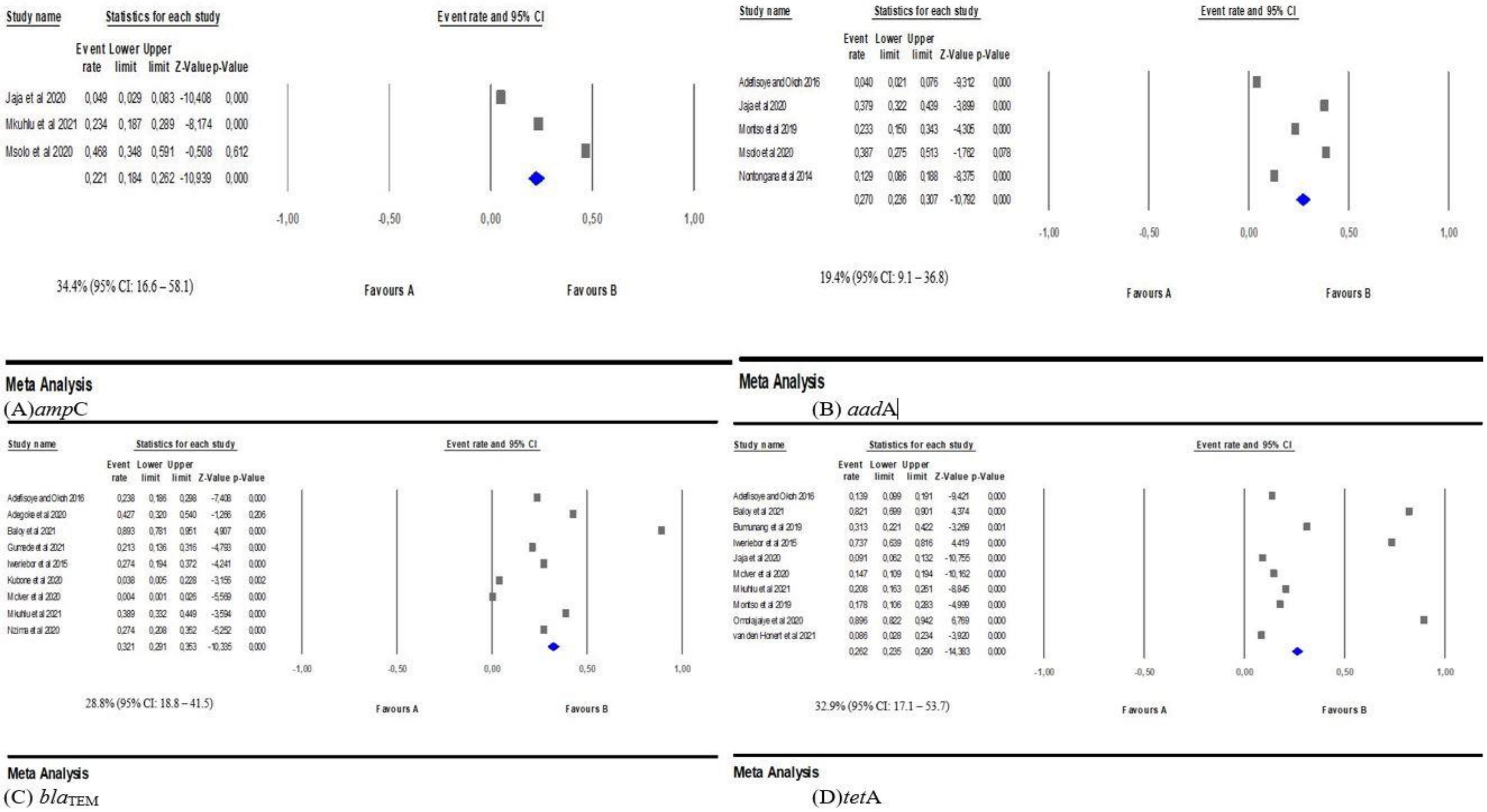

Figure S1. Forest plot showing the pooled estimates of (A)*amp*C, (B) *aad*A, (C) *bla*TEM and D) *tet*A resistance genes from *E. coli*. The squares demonstrate the individual point estimate. The diamond at the base indicates the pooled estimates from the overall studies.

AIMS Press

© 2023 the Author(s), licensee AIMS Press. This is an open access article distributed under the terms of the Creative Commons Attribution License (http://creativecommons.org/licenses/by/4.0)
